# Down-regulation of tumor endothelial marker 8 suppresses cell proliferation mediated by ERK1/2 activity

**DOI:** 10.1038/srep23419

**Published:** 2016-03-21

**Authors:** Chuangjie Cao, Zhuo Wang, Leilei Huang, Lihong Bai, Yuefeng Wang, Yingjie Liang, Chengyun Dou, Liantang Wang

**Affiliations:** 1 Department of Pathology, First Affiliated Hospital, Sun Yat-Sen University, Guangzhou 510080, China; 2 Department of Respiratory, First Affiliated Hospital, Sun Yat-Sen University, Guangzhou 510080, China; 3 Department of Hepatology, Qilu Hospital of Shandong University, Jinan 250012, China

## Abstract

Tumor endothelial marker 8 (TEM8) was recently suggested as a putative anti-tumor target in several types of human cancer based on its selective overexpression in tumor versus normal endothelial cells. The objective of this study was to detect the potential functions of TEM8 in osteosarcoma. Overall, TEM8 was mainly located in cytoplasm and was up-regulated in osteosarcoma compared to benign bone lesions and adjacent non tumor tissue (ANT). High TEM8 expression group had a significant lower overall survival rate than that in the low TEM8 expression group. TEM8 knock-down by siRNA or shRNA results in significant reduction of osteosarcoma cell growth and proliferation both *in vitro* and *in vivo*. Ablation of TEM8 led to increasing of p21 and p27 and suppression of cyclin D1 mediated by Erk1/2 activity. These findings suggest that down-regulation of TEM8 play an important role in the inhibition of tumorigenesis and development of osteosarcoma.

Osteosarcoma, a malignant primary tumor, is the most common primary bone tumor in childhood and adolescence^1^,^2^. Osteosarcoma originates from primitive bone-forming mesenchymal stem cells^3^ and characterizes as rapid growth and high metastatic potential. Lung and bone are two main prevalent sites of metastases of osteosarcoma^4^. Despite increasing understanding of the pathogenesis of osteosarcoma and advanced modern therapy, 40–50% of patients will develop metastases with dismal prognosis^5^. Several genes have been found to be associated with osteosarcoma^6^, which may contribute to diagnosis and targeted therapy.

Tumor endothelial marker 8 (TEM8), also named anthrax toxin receptor 1 (ANTXR1^7^), is originally identified based on its over-expression in the endothelial cells (ECs) that line the tumor vasculature of human colorectal cancer^8^. Genetic disruption of TEM8 results in impaired growth of human tumor xenografts of multiple cancer types^9^ including melanoma, breast, lung, and colon cancer. Antibodies developed against the TEM8 extracellular domain displayed broad antitumor activity^9^. Furthermore, an engineered antibody-like molecule (TEM8-Fc) showed antitumor activities in LS-180, MCF-7, and HepG2 cells^10^. However, little is known about TEM8 expression and its function in osteosarcoma.

In the present study, we demonstrated that TEM8 expression is up-regulated in samples with osteosarcoma. High TEM8 expression group had a significant lower overall survival rate than that in the low TEM8 expression group. TEM8 knock-down by siRNA or shRNA resulted in significant reduction of osteosarcoma cell growth and proliferation. Importantly, ablation of TEM8 led to increasing of p21 and p27 and suppression of cyclin D1 mediated by Erk1/2 activity.

## Results

### Over-expression of TEM8 in osteosarcoma tissue

**Figure 1:**
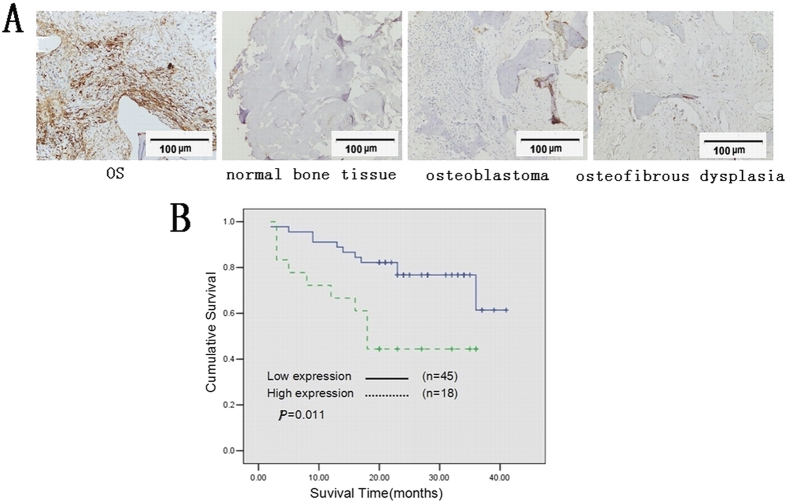
Overexpression of TEM8 in osteosarcoma tissues. (**A**) Immunohistochemistry of TEM8 in tissue specimens: TEM8 protein was upregulated in osteosarcoma tissue compared with normal bone tissue, benign bone tumors (200* magnification). (**B**) Kaplan-Meier curves with univariate analyses (log-rank) for osteosarcoma patients with low versus high TEM8 expression. *p* value was calculated by the log-rank test.

To investigate the expression pattern in clinical samples of osteosarcoma, immunohistochemistry, western blot and qRT-PCR analyses were used. Tumor samples from 63 osteosarcoma patients were collected for immunohistochemistry. TEM8 was mainly located in cytoplasm with minor nuclei distribution. The mean density of TEM8 in 63 osteosarcoma patients were 0.045 (0.029–0.085). The high expression level of TEM8 was assumed to be a mean TEM8 expression density ≥0.08. A mean expression density <0.08 indicated a low level of TEM8 expression. A low level of TEM8 expression 0.005 (0.000–0.054) was found in the control group including 18 cases of normal bone tissues, 10 cases of osteosblastoma, 10 cases of osteoma, 4 cases osteoid ostoma and 8 cases of tumor-like bone lesions (osteofibrous dysplasia and fibrous dysplasia Fig. 1A). The relationship between TEM8 expression and the clinicopathological features of osteosarcoma was summarized in Table 1. Among these 63 patients, there was a statistically significant difference in Enneking staging system^11^ and TEM8 expression (*P* = 0.018). The significant difference also exist between TEM8 and hematogenous metastasis (*P* = 0.011) (Table 1). Moreover, Kaplan-Meier analysis indicated that osteosarcoma patients with the high TEM8 expression had a significant lower overall survival rate compared to those with the low TEM8 expression (Fig. 1B). The cumulative 40-month survival rate was 75.6% in the low-TEM8 protein expression group (n = 45, bold line), while it was only 44.4% in the high-TEM8 protein expression group (n = 18, dotted line). In addition, univariate analyses indicated that high TEM8 expression (*P* = 0.019), metastasis status (*P* < 0.001) and Enneking staging (*P* = 0.002) were significantly associated with shorter overall survival rates (Table 1).

**Figure 2:**
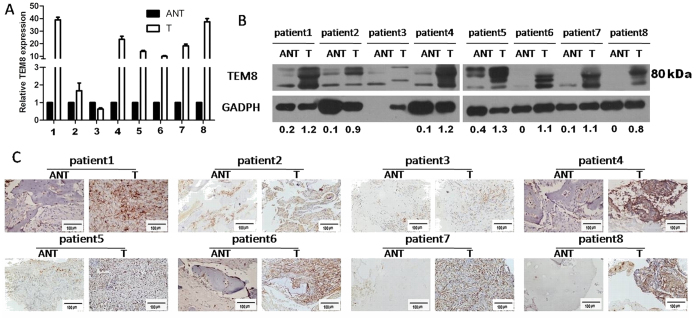
TEM8 expression in ajacent nontumor tissue(ANT) and osteosarcoma(T). TEM8 expression was elevated in at least six of eight cases of osteosarcoma compared with that in ANT by (**A**) RT-PCR, (**B**) western blot(the ratio of TEM8/GAPDH was indicated below) and (**C**) IHC(×200 magnification).

Furthermore, 8 paired osteosarcoma tissue and adjacent non-tumor tissue (ANT) were analyzed. It revealed that mRNA and protein levels were elevated in six tumor tissues compared to their respective ANT tissues (Fig. 2A–C).

### TEM8 knock-down reduced osteosarcoma cell growth *in vitro*

**Figure 3:**
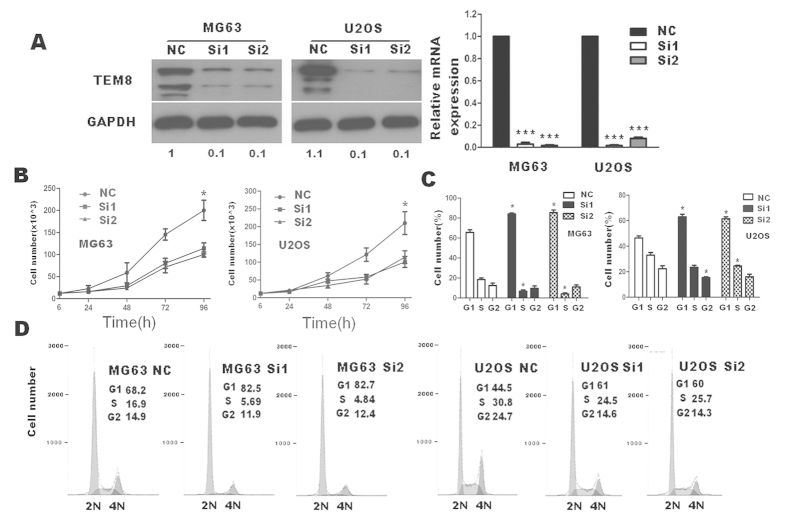
Effects of TEM8 siRNA on cell proliferation and cell cycle distribution of U2OS and MG63 cells *in vitro*. (**A**) TEM8 expression reduced in U2OS and MG63 transfected with TEM8-siRNA (western blot: the ratio of TEM8/GAPDH was indicated below); (**B**) Down regulation of TEM8 reduced the capability of cell proliferation by trypan blue assay. (**C,D**) Down regulation of TEM8 dramatically blocked cell cycle progression, as shown by an obvious increased G1-phase cell population and a decreased G1–S transition. *p < 0.05; ***p < 0.001.

To investigate TEM8 function, siRNA techniques were used to inhibit the endogenous expression of TEM8. SiRNA could successfully down regulate TEM8 expression (Fig. 3A). The migration and invasion ability did not change obviously between scramble siRNA and ablated siRNA through matrigel invasion and migration Assay (Figure S1). The apoptosis assay did not show much significance in both early and late apoptosis through flow cytometry (Figure S2).

Strikingly, TEM8 knock-down significantly attenuated cell proliferation rate through through trypan blue exclusion assay (Fig. 3B) and MTT analysis (Figure S3A). In addition, cell cycle analysis (Fig. 3C,D) showed that cells in the G1 phase were obviously higher in siRNA groups than control groups through cell cycle analysis.

**Figure 4:**
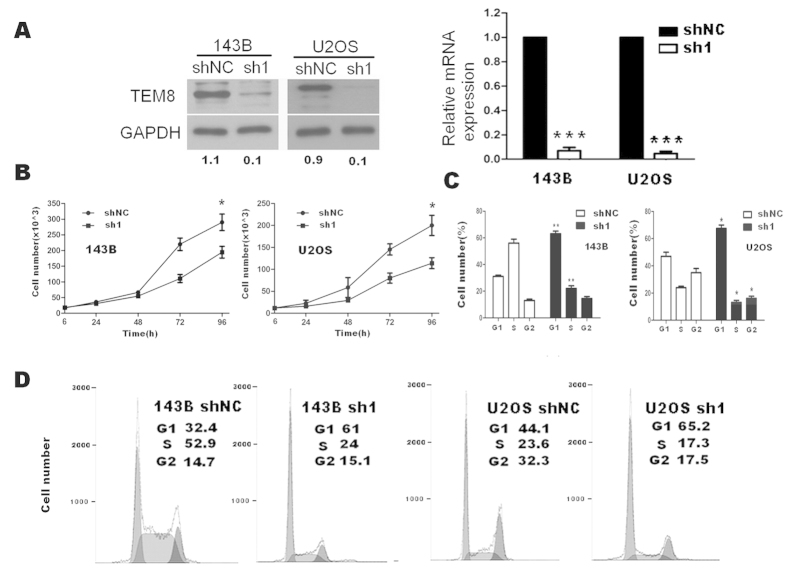
Effects of TEM8 shRNA1 on cell proliferation and cell cycle distribution of 143B and U2OS cells *in vitro*. (**A**) TEM8 expression reduced in 143B and U2OS transfected with TEM8-shRNA1 (western blot: the ratio of TEM8/GAPDH was indicated below); (**B**) Down regulation of TEM8 reduced the capability of cell proliferation by trypan blue assay. (**C,D**) Down regulation of TEM8 dramatically blocked cell cycle progression, as shown by an increased G1-phase cell population and a decreased G1–S transition. *p < 0.05; ***p < 0.001.

We successfully screened shRNA1 to stably down regulate the expression of TEM8 (Fig. 4A), and also found that ablation of TEM8 greatly attenuated the proliferation(Fig. 4B and Figure S3B) and induced G1 to S cell cycle inhibition(Fig. 4C,D) in osteosarcoma cells.

### TEM8 knock-down attenuated ERK1/2 phosphorylation, down-regulated cyclinD1 expression and upregulated p21 and p27 in osteosarcoma

**Figure 5:**
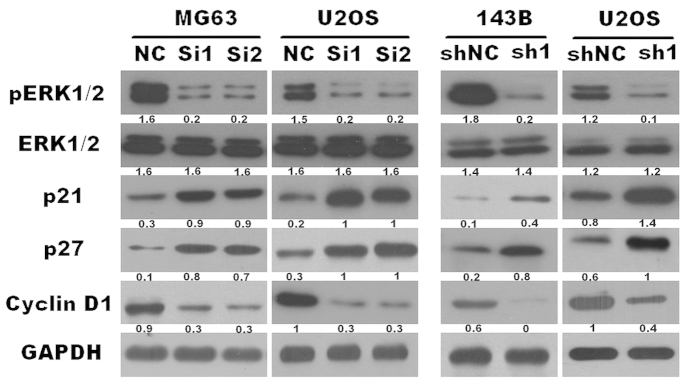
Down regulation of TEM8 inhibited the activation of ERK1/2, expression of cyclinD1, and increased the expression of p21 and p27 (the ratio of molecule/GAPDH was indicated below).

As our previous research demonstrated^12^, ERK1/2 signaling pathway was closely correlated in the development of osteosarcoma, and anthrax lethal factor induced tyrosine/threonine phosphorylation of MAPKs in cultured macrophage^13^. Therefore we attempt to determine whether TEM8 (also named anthrax toxin receptor 1^7^) mediated ERK1/2 signaling. Our results showed that TEM8 knock-down by both siRNA and shRNA reduced the level of phosphorylated-p44/42 protein without altering the total p44/42 level (Fig. 5). TEM8 ablation decreased cyclinD1 protein level (Fig. 5). Interestingly, we found that the expression levels of p21 and p27 were drastically increased in TEM8 silenced cells (Fig. 5).

### TEM8 knock-down increased p21, p27 and suppressed cyclin D1 expression mediated by ERK1/2 activity

**Figure 6:**
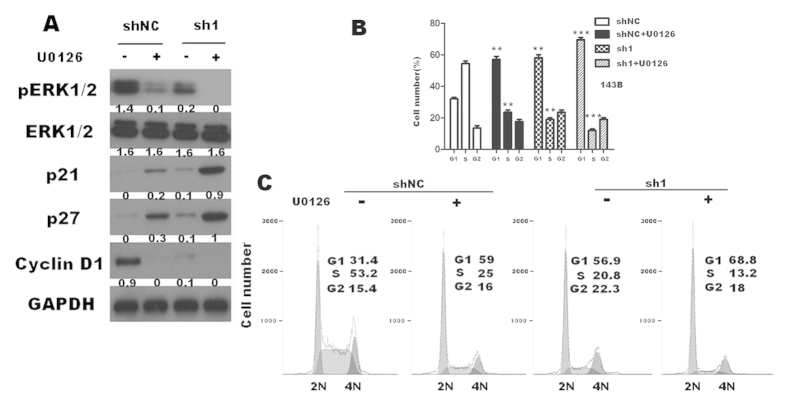
U0126 (40μM) further reduced the proliferation of osteosarcoma cells. (**A**) U0126 enhanced the effects of TEM8-sh1 reducing cyclinD1 expression , and further increased p21 and p27 expresssion in 143B sh1 cell line(the ratio of molecule/GAPDH was indicated below) (**B**,**C**) U0126 further inhibited G1 to S phase transition of 143B sh1 cells.

To further investigate the mechanism of TEM8 mediated proliferation, we used U0126 (a highly selective and potent inhibitor of pERK1/2) on TEM8-143B-sh1 and TEM8-143B-shNC cells. We found that the expression levels of cyclinD1 were significantly reduced in TEM8-shRNA1-transduced cells, accompanied by its further decreasing under the effect of U0126 (Fig. 6A). At the same time, p21 and p27 expression were further increased as U0126 was added (Fig. 6A). Accordingly, cell cycle analysis showed further significant increasing in G1 phase as U0126 was added in sh1 cells (Fig. 6B,C).

### TEM8 knock-down attenuated xenograft tumor growth

**Figure 7:**
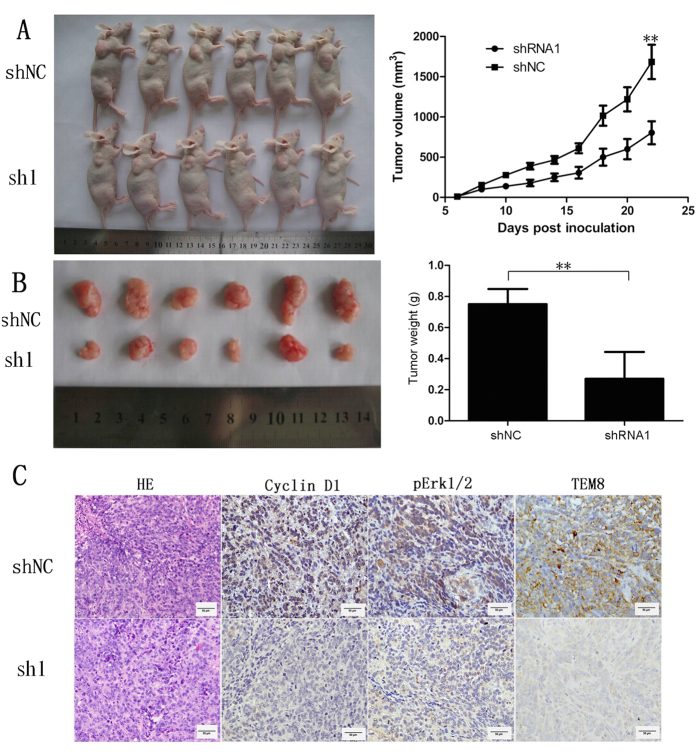
Effects of TEM8 silencing on osteosarcoma xenograft tumor growth *in vivo* significantly suppressed tumor growth. (**A**) sh1-TEM8 significantly suppressed tumor growth of 143B cells implanted subcutaneously in BALB/c nude mice compared with the shNC group (*P* < 0.01; **A**) and significantly reduced tumor weight (*P* < 0.01; **B**). (**C**) Histological features of tumor xenograft by HE, cyclin D1, pERK1/2 and TEM8 immunohistochemistry staining. 143B-shRNA1 tumors displayed remarkably lower cyclin D1, pERK1/2 and TEM8 expression compared with the control group(×400 magnification).

To determine whether TEM8 affects the tumorigenicity of osteosarcoma cells *in vivo*, shTEM8 143B or empty vector-143B cells were subcutaneously injected into nude mice. Tumors formed from 143B-shRNA1 cells grew more slowly (Fig. 7A) and weighed substantially lighter (Fig. 7B) than those formed from 143B-shNC cells. Moreover, 143B-shRNA1 tumours displayed remarkably lower cyclin D1, pERK1/2 and TEM8 expression compared with the control group (Fig. 7C).

## Discussion

Some studies have identified genes encoding tumor endothelial markers (TEMs) that displayed elevated expression during tumor angiogenesis. TEM8 is one of type I membrane proteins, with 96% amino acid identity between the human and mouse proteins, which have been extensively studied for their role as anthrax toxin receptors, but with a still elusive physiological function. The expression pattern of TEM8 was especially intriguing in that it is the only human TEM characterized thus far that shows no detectable mRNA expression in either the corpus luteum or healing wounds, suggesting that this gene may be highly specific to tumor angiogenesis and not required for “normal” adult angiogenesis^8^,^14^. Recent evidence suggested that angiogenesis played a pivotal role in the development of hepatocellular carcinoma cells, thus the therapy strategy targeting antiangiogenesis has been regarded as promising method for hepatocellular carcinoma therapy^15^. Moreover, the recombinant adenovirus encoding TEM8 modified dendritic cells may induce antitumor immunity by disrupting tumor vasculature and may be used to influence tumor development in clinical applications^15^. Other research proved that TEM8 protein interacted with the C5 domain of collagen a3, which was also preferentially expressed in tumor endothelium. Then TEM8 expression stimulated endothelial cell adhesion and migration^16^,^17^. Yet, no functional significance has been assigned to TEM8 as a regulator of osteosarcoma biology.

Gloria Bonuccelli *et al*.^18^. reported 3 isoforms tested by westernblot: near 80 kDa, near 55 kDa and near 45 kDa. Besides, including isoform 1, 2 and 3, Micaela Vargas *et al*.^19^. detected isoform 4 and isoform 5 of TEM8 in human tissues by nested PCR. However, which isform played what biological function remained illusive. Our western blot demonstrated that dominant TEM8 were near 80 kDa both in clinical samples and osteosarcoma cell lines, which was somewhat in accordance with the study of Akash Nanda *et al*.^20^ who revealed an 80–85 kDa doublet in 293 cells transfected with full length TEM8. Therefore, we thought that long isoform of TEM8 (isoform 1) probably plays a dominant role in osteosarcoma compared with other isoforms.

Singh *et al*. have found TEM8 upregulated in androgen-independent prostate cancer cells, although further researches did not involved in the function of TEM8 about tumor cells^20^. Interestingly, we found that osteosarcoma cells could express TEM8 protein. And the expression is similar. In the present study, we demonstrated that TEM8 was mainly located in cytoplasm and was up-regulated in osteosarcoma compared to ANT. TEM8 was up-regulated in osteosarcoma and its up-regulation was associated with the lower survival rate of osteosarcoma patients suggesting that TEM8 might be involved in the progression of osteosarcoma.

Kylie A. Hotchkiss *et al*.^17^ found that TEM8-expressing endothelial cells migrated faster than control cells in a monolayer denudation assay. Our findings did not show any difference between TEM8 siRNA group and control group in migration and invasion. And neither their results^17^ nor our results did TEM8 endow endothelial cells or osteosarcoma cells with effect on an apoptotic stimulus. Genetic disruption of TEM8 resulted in impaired growth of human tumor xenografts of diverse origin and antibodies developed against the TEM8 extracellular domain displayed broad antitumor activity^9^. Dendritic cells transduced with TEM8 recombinant adenovirus inhibited cells growth^15^. Furthermore, an engineered antibody-like molecule (TEM8-Fc) showed antitumor activities in LS-180, MCF-7, and HepG2 cells^10^. We found that down regulation of TEM8 attenuated osteosarcoma cell growth *in vitro* which was accompanied by cell cycle G1 phase to S phase arrest. The knockdown efficiency was about 90% repression. However, there was no more than 50% of cell proliferation reduced. This indicated that TEM8-ERK1/2-cyclin D1 was not the only mechanism which could modulate the proliferation of osteosarcoma. Probably other signaling pathway functions compensably in the proliferation of osteosarcoma. And this needed further investigation.

The progression of osteosarcoma is linked to the activation of many signaling pathways, one of which is MAPK, MEK and ERK1/2 signaling^21^. ERK1/2 activity plays an important role in tumorigenesis via the regulation of a variety of biologic processes, such as apoptosis, proliferation and migration through induction of the cyclin dependent kinase (CDK) inhibitors, including p21 and p27^22^,^23^,^24^. As our previous study^12^ demonstrated, ERK1/2 played an important role in the development of osteosarcoma. In the present study, we found that pERK1/2 was decreased in TEM8-ablated cells, suggesting that TEM8-induced proliferation and tumorigenesis may be due to modulation of ERK1/2 activity.

Cyclins are the key constituents of the cell cycle machinery. They could combined with their respective cyclin-dependent serine/threonine protein kinases and formed the holoenzymes that phosphorylate different sets of target proteins thereby driving the cell through consecutive phases of the cycle^25^. The cellular abundance of cyclin D1, as of other cyclins, is regulated by their synthesis and degradation. Cyclin D1 as a key regulatory proteins controlled the transition from G1 to S phase by binding to CDK4/CDK6 and CDK2^26^. The synthesis of cyclin D1 is induced by growth factors that stimulate the MAPK/ERK (Ras-Raf-MEK-ERK) pathways^27^. At the same time, we found that TEM8 ablation decreased cyclinD1 protein level in osteosarcoma cells. It suggested that cyclinD1 played an important role in TEM8 prompting osteosarcoma proliferation.

The protein p21^WAF1^ is a cyclin-dependent kinase inhibitor (CKI) which binds and inhibits the activity of cyclin-CDK1, cyclin-CDK2, and cyclin-CDK4/6 complexes. Other research showed that p21^WAF1^ was a checkpoint regulator of cell cycle progression at G1 and S phase^28^. Meanwhile, we showed that the mechanism of TEM8-mediated proliferation was linked to alternations in the expression of the cell cycle inhibitors p21 and p27 as well as the CDK regulator cyclinD1. This was another powerful evidence to prove the function of TEM8 in osteosarcoma proliferation. In addition, pretreatment with U0126 on TEM8-shRNA cells further upregulated P21and P27 and downregulated cyclin D1 demonstrated the important role of ERK1/2 in this process. U0126 is a potent and selective inhibitor of MEK1 and MEK2 kinases. U0126 has been reported as a specific inhibitor of the ERK1/2 signaling pathway. It has been widely used as an inhibitor for the Ras/Raf/MEK/ERK signaling pathway with over five thousand references on the NCBI PubMed database. Thus, we thought that TEM8 might regulate osteosarcoma cell growth/proliferation at least partly though ERK1/2 activity. From the results above, TEM8 plays an important role in regulating the proliferation of osteosarcoma. However, which specific isoform dominant an specific physiological role or whether each one have the same functions in osteosarcoma was still unclear. To solve this problem, specific antibody and specific siRNA against different isoforms must be generated. These need further investigation.

In summary, our results suggest that TEM8 plays an important role in human ostesarcoma progression. Full understanding of the precise role of TEM8 in human cancer may provide the opportunity to develop a novel therapeutic strategy by suppressing expression of TEM8 in ostesarcoma.

## Methods

### Clinical samples and characteristics

A total of 78 paraffin-embedded primary osteosarcoma specimens were analyzed. All cases had been clinically and histopathologically diagnosed at the First Affiliated Hospital of Sun Yat-Sen University (Guangzhou, China) from January 2010 to July 2013. The histology of the disease was determined according to the criteria of the World Health Organization. For research purposes, prior patient (or guardian) consent and approval of the Institutional Research Ethics Committee were obtained. Among 78 cases, only 63 cases had full detailed clinical data, including age, sex, location, metastasis status, tumor size, Enneking staging and survival status. Eight fresh samples paired adjacent non-tumor tissues (ANT) from surgery were frozen and stored at −80 °C for western blot and RNA examination. Tissue specimens in research were approved by research ethics committee of the First Affiliated Hospital at Sun Yat-sen University, and all subjects confirmed that informed consent were obtained from the First Affiliated Hospital at Sun Yat-sen University. The use of tissue specimens were carried out in accordance with the approved guidelines of the First Affiliated Hospital at Sun Yat-sen University.

### Immunohistochemistry

Four-μm-thick sections were deparaffinized, rehydrated in serially graded ethanol, heated in citric buffer (pH 6.0) once for 20 min in a microwave oven for antigen retrieval, and blocked with 3% hydrogen peroxide for 15 min. The samples were then labeled with anti-TEM8 antibody (1:300, Abcam, #21270), anti-cyclin D1 antibody (1:200, cell signaling technology) and anti-pERK1/2 antibody (1:200, cell signaling technology) at 4 °C overnight. The next day, after washing with phosphate-buffered saline (PBS), the sections were incubated with EnVision-HRP secondary antibody (DAKO, Carpinteria, CA) for 30 min at 37 °C in a water bath, washed with PBS, stained with 0.5% diaminobenzidine and counterstained with Mayer’s hematoxylin, then air dried, and mounted with resinene.

### Evaluation of Immunohistochemistry

The immunohistochemical staining in osteosarcoma and ANT were subjected to microscope and image analysis (Nanozoomer, Hamamatsu, Japan). Briefly, after IHC staining, if a cell or tissue was stained from light yellow to brown, it would be recorded as positive immunostaining. The areas from both cancer and its adjacent normal tissue were selected for analysis. The intensity of the staining signal was measured and documented using the Image-Pro Plus 6.0 image analysis software (Media Cybernetics, Inc. Silver Spring, MD USA). The mean densitometry of the digital image (×200 or ×400) is designated as representative IHC staining intensity. The signal density of tissue areas from three randomly selected visions were counted blindly and subjected for statistical analysis. The positive regions were analyzed in Image-Pro Plus 6.0 to determine the integral optical density and area. The average optical density was calculated. The average of three optical density values was determined to represent the expression intensity in the section.

### RNA extraction and quantitative qRT-PCR

Total RNA from fresh tissues was extracted using Trizol reagent (Invitrogen, Carlsbad, CA) according to the manufacturer’s instruction and was extracted using RNApure Tissue Kit (Cwbiotech, Beijing, China). One μg of RNA from each sample was used for cDNA synthesis (Roche, Basel, Switzerland) and quantitative real-time PCR analysis (Roche, Basel, Switzerland). The primers for amplification were: TEM8^29^ sense and antisense were labeled in supplemental Table 1, GAPDH (supplemental Table 1) was used as an internal control.

### Cell lines

Four cell lines including U2OS, MG63, 143B and 293T were obtained from our laboratory cell bank, Guangzhou, China. U2OS and 293T were grown in Dulbecco’s modified Eagle’s minimal essential medium (DMEM, Gibco, Carlsbad, CA). MG63 and 143B were cultured in RPMI 1640 medium (Gibco, Grand Island, NY). All media were supplemented with 10% fetal bovine serum (FBS, Hyclone, Tauranga, New Zealand), 100 ug/ul streptomycin and 100 ug/ul penicillin in a 37 °C incubator containing 5% CO_2_.

### Small interfering RNA transfection and the generation of stably transfected cell lines

Double-stranded small interfering RNA targeting TEM8 (50 nM, synthezed by Ribobio (Guangzhou, China); siTEM8 RNA and scrambled control siRNA (supplemetal Table 1) were transfected into U2OS and MG63 cell lines using Lipofectamine RNAiMAX (Invitrogen) according to manufacturer’s instruction. The TEM8 short hairpin RNAs (shRNAs) was purchased from Sigma-Aldrich according to catalog SHCLND-NM_018153 including shRNA 1–5 and shNC. The shTEM8 plasmid or empty vector was co-transfected into 293FT cells along with the retroviral packaging vector. After transfection, the supernatants were harvested to infect U2OS and 143B cells, and the stably transfected cells were selected with puromycin.

### Western Blot

Cells were lysed in RIPA buffer, agitated for 20 minutes at 4 °C, sonicated for 15 seconds using sonic oscillator and centrifuged at 12,000 rpm for 15 minutes. The concentration of total proteins was determined using BCA method. Total proteins (30 μg) in equal volume were then denatured and loaded on 10% SDS polyacrylamide gels for separation. The proteins were transferred onto polyvinylidene difluoride membranes that were subsequently blocked in 8% nonfat milk in TBST. The membranes were incubated with primary antibodies including anti-TEM8 (Abcam, #21270), anti-p21^Cip^(Santa Cruz, sc-6246), anti-p27^Kip^(BD, 610242), anti-p44/42 MAPK (ERK1/2), anti-phospho-p44/42 MAPK (pERK1/2), and anti-GAPDH (Cell Signaling Technology, Danvers, MA) at 4 °C overnight. After washing 3 times with TBST, the membranes were probed with secondary antibody HRP-conjugated goat anti-rabbit (1:5000, Cell Signaling Technology) or goat anti-mouse (1:2000, CST) and visualized by enhanced chemiluminescence. The tags of TEM8 have 3 isoforms tested by westernblot: near 80 kDa, near 55 kDa and near 45 kDa^18^.The full-length TEM8^16^ is found to express near 80 kDa. Tags near 80 kDa is a dominant isoform both in fresh tissues and osteosarcoma cell lines.

### Invasion and Migration Assay

For invasion assay, U2OS and MG63 cells transfected with TEM8 siRNA or Scrambled siRNA were trypsinized and resuspended to a density of 1 × 10^5^/ml in serum-free medium. A 200-ul cell suspension was plated into the top side of polycarbonate Transwell filter coated with 30 mg/cm^2^ of Matrigel in the upper chamber of the BioCoatTM Invasion Chambers (BD, Bedford, MA). Each well was sustained 400 ul DMEM medium or RPMI 1640 medium respectively. After incubation at 37 °C for 36 h, cells on the upper chamber were removed by wiping gently with cotton swabs. Cells on the lower membrane surface were fixed with 1% paraformaldehyde for 20 minutes, stained with 0.3% Crystal Violet solution for 20 minutes, and then counted (five high-power fields per chamber). The migration assay was performed by the same procesure as the invasion assay except that no matrigel was added to the transwell membrane. Both experiments had three independent replicates and the data were presented as mean ± standard deviation (SD).

### Cell proliferation analysis

Cell proliferation analysis was conducted using trypan blue assay and 3-(4,5-dimethylthiazol-2-yl)-2,5-diphenyl tetrazolium bromide(MTT) kit(Keygen, Nangjing, China). In brief, after transfection with TEM8 siRNA and siNC for 6 h, the cells were harvested for plating in sterile 12-well plates 24 h after transfection(0 h). U2OS cells and MG63 cells were plated of 1 × 10^4^ in each well. Cells were harvested by trypsinization, stained using a 0.4% Trypan Blue solution at 6, 24, 48, 72 and 96 h and the total volume was 1 ml per well. Cells were counted in an optic microscope. For stably transfected shRNA1 or shNC U2OS and 143B, cells were seeded on 12-well plates of 1 × 10^4^ cells per well. Cells were harvested by trypsinization, stained using a 0.4% Trypan Blue solution at 6, 24, 48, 72 and 96 h and the total volume was 1 ml per well. Cells were counted in an optic microscope. In brief, after transfection with TEM8 siRNA for 6 h, the cells were harvested for plating 24 h after transfection(0 h). U2OS cells (1 × 10^3^) and MG63 cells (1 × 10^3^) were plated onto 96-well plates with medium containing 8% FBS. Cell proliferation was determined at 6, 24, 48, 72 and 96 h using the MTT assay. For stably transfected shRNA1 and shNC U2OS, cells were seeded on 96-well plates at 1 × 10^3^ cells per well. At 6, 24, 48, 72 and 96 h, cell proliferation was determined by MTT assay. The absorbance was measured with 570 nm wavelength. This experiment had three independent replicates.

### Cell cycle analysis

U2OS and MG63 cells (3 × 10^5^) were seeded in each well of six-well plates and incubated overnight until 30–50% confluent, then transfected withTEM8 siRNA or scrambled siRNA for 6 h. The cells were harvested for test at 24–48 h after transfection. For stably transfected with shRNA1 and shNC, cells were incubated to reach 90% confluent until harvested. Then cells were washed with cold PBS, fixed with 70% ethanol for overnight at 4 °C, and conducted using cell cycle analysis kit(Keygen, Nangjing, China). Finally, cells were analyzed by a FACScan flow cytometer (Becton Dickinson, Franklin Lakes, NJ) with Cell Quest Pro software. This experiment had three independent replicates.

### Apoptosis assay

After 24 h of TEM8 siRNA transfection, U2OS and MG63 cells were immediately serum-starved (time 0 h) and incubated. Both floating and adherent cells were collected after 24 h of starvation, and washed once with cold PBS, then resuspended in 500 μl cold Annexin binding buffer containing 5 μl Annexin V-FITC and 5 μl propidium iodide. The cells were incubated for 15 min in the dark at room temperature and analyzed using a FACScan flow cytometer (Becton Dickinson, Franklin Lakes, NJ) with Cell Quest Pro software. The percentage of apoptotic cells was quantified using Cell Quest Pro software. This experiment had three independent replicates.

### *In vivo* tumorigenicity assays

BALB/c-nude immune deficient mice (4 weeks old, 14–16 g) were housed under pathogen-free and 12 h light/dark cycle condition. The mice were randomly assigned into shNC and shRNA1 groups (n = 6 mice/group). 143B cells were trypsinized, washed twice with serum-free MEM and reconstituted at a concentration of 1 × 10^6^ cells/100 μl in serum-free MEM, then inoculated subcutaneously into the right flank of each nude mouse. The mice were monitored daily for adverse affects. Tumor size was recorded on day 6 and calculated every other day, using a digital caliper, and the tumor volume was calculated according to the formula: tumor volume (mm^3^) = length × width^2^ × 0.5. At the end of the experiment, all the mice were sacrificed and the total weights, tumor weights and tumor volumes were recorded. Each tumor were stained of TEM8, cyclin D1, pERK1/2 and HE for immunohistochemistry. All experimental procedures were carried out in accordance to and were approved by the Guidance of Institutional Animal Care and Use Committee of Sun Yat-Sen University.

### Statistical analysis

Significant associations between TEM8 expression and clinicopathological parameters were assessed using one way ANOVA; Data from cell culture were compared using Student’s t test. Overall survival rate for each variable was computed by the Kaplan-Meier method. The log-rank test was used to compare the differences in survival curves. Cutoff values to define high and low expression of TEM8 were chosen on the basis of a measure of heterogeneity with the log-rank test with respect to overall survival. An optimal cutoff value 0.08 was identified. All data were analyzed using SPSS 13.0. Significance levels were reported in the figures. “*” means *P* < 0.05; “**” means *P* < 0.01 and “***” means *P* < 0.001.

## Additional Information

**How to cite this article**: Cao, C. *et al*. Down-regulation of tumor endothelial marker 8 suppresses cell proliferation mediated by ERK1/2 activity. *Sci. Rep*. **6**, 23419; doi: 10.1038/srep23419 (2016).

## Supplementary Material

Supplementary Information

## Figures and Tables

**Table 1:** Clinicopathological Parameters in Osteosarcoma Cases.

Variable	Cases(n)	%	Expression level of TEM8	*P* value	Survival analysis(*P* value) Univariate
Age					
<20	55	87.3	0.049 (0.021–0.083)	0.963	0.235
≥20	8	12.7	0.057 (0.015–0.017)		
Age(years) at diagnosis	6–36 (median 15)				
Sex					
Male	39	61.90	0.041 (0.022–0.090)	0.887	0.960
Female	24	38.10	0.049 (0.033–0.083)		
Location					
Femur	40	63.49	0.063 (0.033–0.089)	0.806	0.489
Tibia	13	20.63	0.045 (0.020–0.120)		
Humerus	5	7.94	0.040 (0.022–0.076)		
Fibula	2	3.17	0.115 (0.019–0.210)		
Costal	1	1.16	0.025		
Radius	1	1.16	0.022		
Vertebrae	1	1.16	0.077		
Metastasis					
Yes	26	46.03	0.075 (0.040–0.098)	**0.48**	**<0.001**
No	37	53.97	0.034 (0.152–0.067)		
TEM8 expression					
Low	45	71.4	0.034 (0.020–0.058)	**<0.001**	**=0.019**
High	18	28.6	0.227 (0.093–0.211)		
Tumor size					
<20 cm^3^	23	36.5	0.040 (0.022–0.060)	0.06	
≥20 cm^3^	40	63.5	0.069 (0.031–0.101)		
Enneking staging					
IA-IIA	31	49	0.035 (0.022–0.076)	**0.019**	**=0.002**
IIB-IIIA	32	51	0.058 (0.032–0.144)		

Expression level of TEM8 were showed as median(low quartile-up quartile).
