# Supplementary material for: Down-regulation of tumor endothelial marker 8 suppresses cell proliferation mediated by ERK1/2 activity
Source: Sci Rep. 2016 Mar 21;6:23419. doi: 10.1038/srep23419 (PMC4800672; doi:10.1038/srep23419)
Supplement: Supplementary Information [file srep23419-s1.pdf]

Down-regulation of tumor endothelial marker 8 suppresses cell proliferation mediated by ERK1/2 activity

Chuangjie Cao[1+], Zhuo Wang[1+], Leilei Huang[1+], Lihong Bai[2+], Yuefeng Wang[1+], Yingjie Liang[1+], Chengyun Dou[3+], and Liantang Wang[1+]*

[1]Department of Pathology, First Affiliated Hospital, Sun Yat-Sen University, Guangzhou, China;
[2]Department of Respiratory, First Affiliated Hospital, Sun Yat-Sen University, Guangzhou, China;
[3]Department of Hepatology, Qilu Hospital of Shandong University, JiNan, China

*Correspondence to: Prof . Liantang Wang, Department of Pathology, First Affiliated Hospital, Sun Yat-Sen University, 72 Zhongshan Road, GuangZhou 510080, People’s Republic of China. Telefax: 020-87331780(*E-mail: wanglt@mail.sysu.edu.cn)

[+]These authors contributed equally to this study.

Supplemental table 1. primers and TEM8 siRNA sequences

	sense	antisense
TEM8	5'-CTG CAC CAC TGG AAT GAA ATC-3'	5'-TGT CTC CTC CTG GCA GAA CTT-3'
GAPDH	5'-GTG GAC CTG ACC TGC CGT CT-3'	5'-GGA GGA GTG GGT GTC GCT GT-3'
siRNA1-TEM8	5'-GGCUAAUAGGUCUCGAGAU dTdT-3'	3'- dTdT CCGAUUAUCC AGAGCUCUA -5'
siRNA2-TEM8	5'-GAUUGCGGACAGUAAGGAU dTdT-3'	3'- dTdTCUAACGCCUGU CAUUCCUA -5'
Scrambled-control siRNA	5'-UUC UCC GAA CGU GUC ACG UTT-3'	5'-ACG UGA CAC GUU CGG AGA ATT-3'

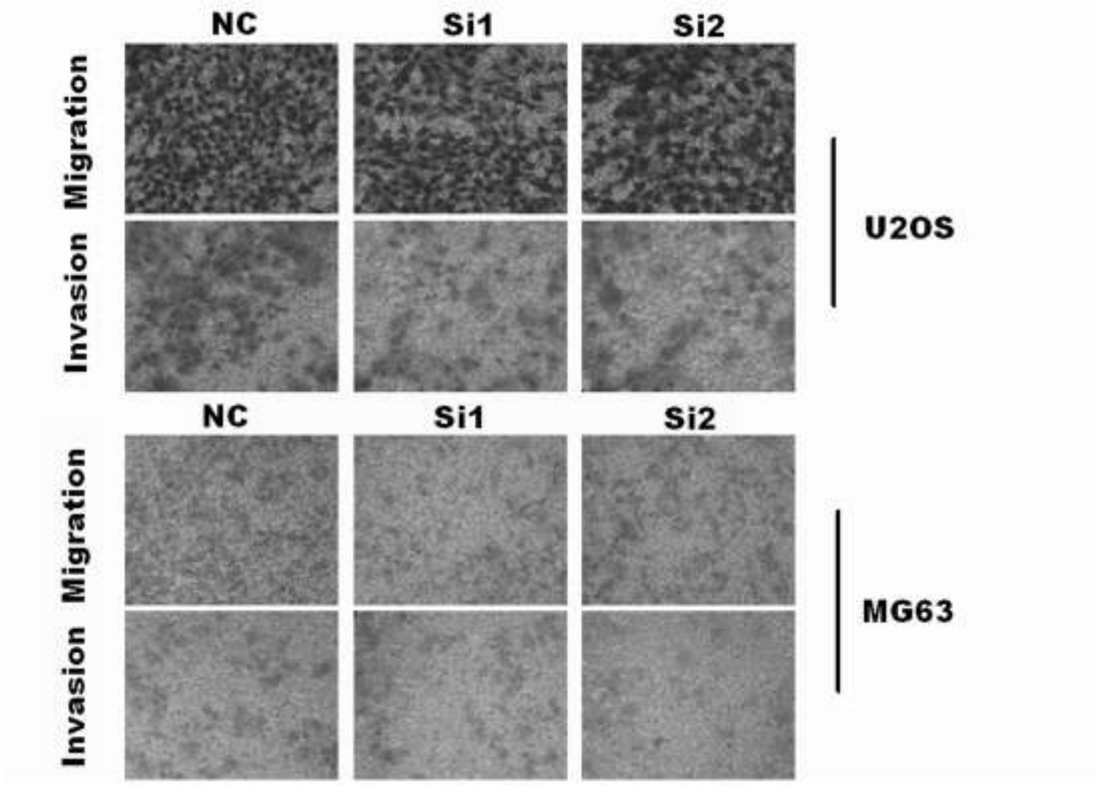

Supplemental Figure 1. TEM8-siRNA-transfected U2OS and MG63 tested by migrartion and invasion assay as cell in siRNA groups had no significant changes compared with NC.

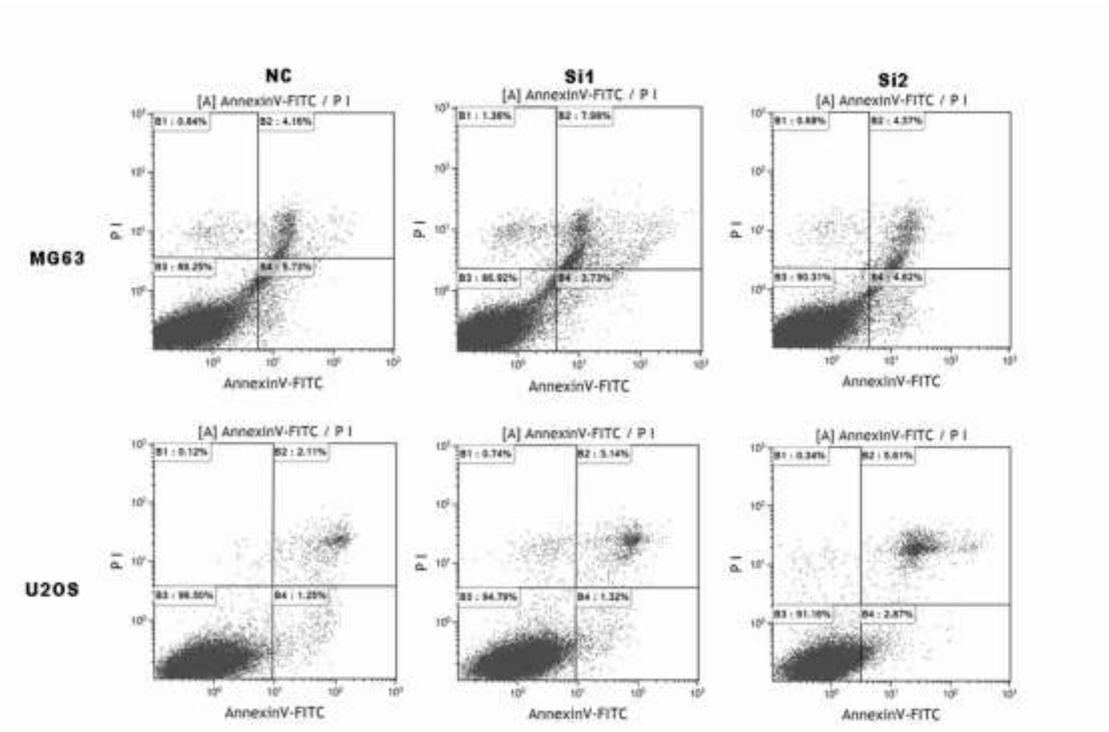

Supplemental Figure 2. TEM8-siRNA-transfected U2OS and MG63 tested by apoptosis assay as cell in siRNA groups had no significant changes compared with NC.

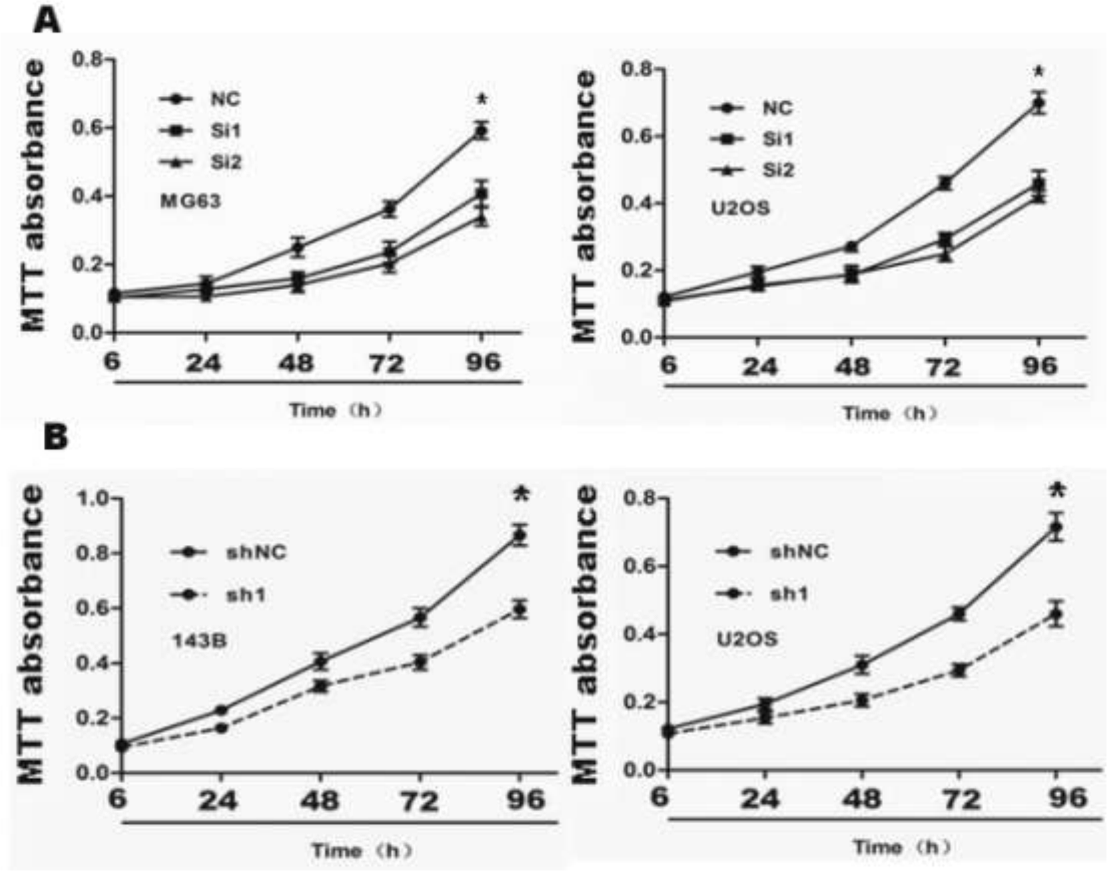

Supplemental Figure 3. A. TEM8-siRNA-transfected U2OS and MG63 tested by MTT assay as proliferat ion rate in siRNA groups were attenuated compared with NC. B. Down regulation of TEM8 in sh1 group reduced the capability of cell proliferation.
